# Evaluation of cross-reactivity to *Taenia hydatigena* and *Echinococcus granulosus* in the enzyme-linked immunoelectrotransfer blot assay for the diagnosis of porcine cysticercosis

**DOI:** 10.1186/s13071-018-3279-5

**Published:** 2019-01-24

**Authors:** Lucho Gomez-Puerta, Ana Vargas-Calla, Yesenia Castillo, Maria Teresa Lopez-Urbina, Pierre Dorny, Hector H. Garcia, Armando E. Gonzalez, Seth E. O’Neal, the Cysticercosis Working Group in Peru

**Affiliations:** 1 School of Veterinary Medicine, Universidad Nacional Mayor de San Marcos, Lima, Peru; 2 Department of Microbiology, Universidad Peruana Cayetano Heredia, Lima, Peru; 3 Institute of Tropical Medicine, Antwerp, Belgium; 4 Center for Global Health Tumbes, Universidad Peruana Cayetano Heredia, Tumbes, Peru; 5 School of Public Health, Oregon Health & Science University - Portland State University, Portland, OR USA

**Keywords:** *Taenia solium*, *Taenia hydatigena*, *Echinococcus granulosus*, Cysticercosis, Enzyme-linked immunoelectrotransfer blot, Porcine, Specificity, Cross-reaction, GP50

## Abstract

**Background:**

*Taenia solium* is an important zoonotic parasite that infects humans as definitive host (taeniasis) and pigs as intermediate host (cysticercosis). Serological diagnosis of porcine cysticercosis is limited to antigen detection using ELISA, which is known to cross-react with other *Taenia* species, and antibody detection using the lentil-lectin glycoprotein enzyme-linked immunoelectrotransfer blot (LLGP EITB), which has not been adequately evaluated for cross-reactivity to other parasites. Field studies suggest that the GP50 diagnostic band of the LLGP EITB may cross-react to *Taenia hydatigena*, a common non-zoonotic parasitic infection of pigs. The objective of this study was to evaluate the specificity of the LLGP EITB assay in pigs infected experimentally with *T. hydatigena* and *Echinococcus granulosus*.

**Results:**

Twelve three-month-old seronegative were divided into two groups; six were each given an oral challenge with a single gravid proglottid of *T. hydatigena* and the other six were each given an oral challenge with 50 gravid proglottids of *E. granulosus*. Serum samples were collected biweekly until 14 weeks when all pigs underwent a detailed necropsy. *Taenia hydatigena* cysticerci were found in two of six pigs from the first group. Four *T. hydatigena-*exposed pigs were seropositive at the GP50-band only on EITB LLGP; two of these had cysts at necropsy while no seronegative pigs had cysts. One *E. granulosus*-exposed pig was positive to EITB LLGP, again with reactivity only to GP50; all six pigs had hepatic echinococcosis on necropsy.

**Conclusion:**

These results provide definitive evidence that the GP50 diagnostic band in pigs cross-reacts with *T. hydatigena*. Evidence of cross-reaction with *E. granulosus* was not conclusive.

**Electronic supplementary material:**

The online version of this article (10.1186/s13071-018-3279-5) contains supplementary material, which is available to authorized users.

## Background

*Taenia solium* is a zoonotic cestode that infects pigs as the intermediate host of the metacestode stage (porcine cysticercosis) and humans as the definitive host of the adult intestinal tapeworm. Humans can also be infected with the metacestode stage resulting in seizures and other neurological manifestations when the parasite encysts in the central nervous system (neurocysticercosis, NCC). The health impact of *T. solium* is substantial in endemic regions around the world where NCC is estimated to be responsible for about 30% of epilepsy [1]. Given the substantial public health harm caused by *T. solium*, the World Health Organization has called for increased efforts towards control and elimination [2].

Serological testing for porcine cysticercosis is used to identify regions where the parasite is endemic and to monitor the progress of control efforts [3–8]. Assays based on antigen (Ag) detection using monoclonal antibodies (mAbs) are available, although these are known to cross-react with other *Taenia* spp. making it difficult to interpret the results from field studies. An antibody (Ab) detection test, the lentil-lectin glycoprotein enzyme-linked immunoelectrotransfer blot assay (LLGP EITB), has been used frequently over the last three decades due to its excellent reported performance characteristics (99% sensitivity and 100% specificity) [9]. However, only a few other parasites (*Ascaris*, *Trichuris* and *Trichinella*) were tested for potential cross-reactivity using sera from controlled experimental exposures [10]. Sera from naturally-infected pigs with *Echinococcus granulosus* and *Fasciola hepatica* were also tested, but dose, timing, and any potential exposure to other parasites could not be verified [10].

The LLGP EITB is based on a semi-purified fraction of seven native *T. solium* glycoprotein antigens (GP50, GP42-39, GP24, GP21, GP18, GP14 and GP13), with the number indicating the molecular weight in kDa [9]. Antibody reaction to one or more of the glycoprotein bands is classically interpreted as evidence of exposure to the metacestode stage of *T. solium*. While it has been hypothesized that pig exposure to or infection with other *Taenia* spp. might result in cross-reactions to one or more of these glycoprotein bands, this issue has remained largely unstudied [11]. One recent study found no evidence of cross-reaction against any of the glycoprotein bands in five pigs that underwent oral challenge with *T. saginata* eggs [12]. In a recent field study in a region where *T. solium* had previously been eliminated [13], we found strong evidence suggesting that exposure of pigs to *T. hydatigena*, a related cestode, could result in cross-reactivity to the GP50 diagnostic band. In a cluster of GP50-positive pigs, the majority were found to be infected with *T. hydatigena* while none was infected with *T. solium* [14]. In addition, adult *T. hydatigena* worms were found in resident dogs whereas no adult *T. solium* worms were found among resident humans. The objective of this study was to verify whether cross-reaction to GP50 occurs in sera from pigs exposed in experimental conditions to *T. hydatigena* and *E. granulosus*, two related cestodes that are frequently co-endemic in regions where *T. solium* transmission occurs.

## Methods

*Taenia hydatigena* and *E. granulosus* adult stage tapeworms were obtained from naturally-infected dogs in the highlands of Cusco, Peru, using arecoline hydrobromide purgation [15]. Intact tapeworms were stored at 4 °C in preservation medium comprised of 25% glycerol, penicillin (1000 U/ml), gentamicin (100 g/ml), streptomycin (1 mg/ml), and amphotericin B (20 g/ml), then transported to the Veterinary School at Universidad Nacional Mayor de San Marcos (Lima, Peru) where the species was determined based on morphological features, including characteristics of the rostellar hooks. Intact gravid proglottids were removed and preserved for a period of 17 days until use in the experimental infection.

We obtained 12 three-month-old Landrace piglets from a commercial farm in Lima, a region where *T. solium* is not endemic. The pigs were verified to be serologically negative for *Taenia* spp. Ag and for Ab against *T. solium* using B60/B158 ELISA [16] and LLGP-EITB [10], respectively. The pigs were then divided into two equal groups of six pigs each housed in separate corrals. In the first group, each pig was given an oral challenge with a single gravid proglottid of *T. hydatigena*. The proglottid was mixed into a mush of oatmeal and plantain, which was formed into a ball and then fed directly to the pig. In the second group, each pig was given an oral challenge with 50 gravid proglottids of *E. granulosus* prepared in the same manner. The pigs were then monitored for a period of 14 weeks in order to allow infecting larvae to mature, at which point the pigs were then humanely euthanized and examined by necropsy.

We took biweekly blood samples all pigs until week 14 when the pigs were euthanized and dissected. Whole blood was allowed to clot and was then centrifuged to separate sera. Serum samples were stored at -20 °C, and later analyzed by LLGP EITB for the presence of Ab and by B158/B60 Ag-ELISA for the presence of *Taenia* spp. antigens [10, 16]. An optical density ratio of > 1 was considered positive on the Ag-ELISA. All pigs were anesthetized after 14 weeks using intramuscular ketamine (20 mg/kg) and xylazine (2 mg/kg), then euthanized by intravenous sodium pentobarbital (60 mg/kg). We systematically dissected each carcass using fine cuts less than 0.5 cm to inspect for the presence of metacestode infection. The dissection included all skeletal muscle tissue, heart, liver, lungs, esophagus and intestines. All suspected viable, degenerating, or calcified cysts were collected and stored in 70% ethanol for subsequent molecular analysis. We used polymerase chain reaction (PCR) to amplify a 392-bp fragment of the cytochrome *c* oxidase subunit 1 gene (*cox*1) using primers JB3 and JB4.5 [17, 18]. We then sequenced the PCR products using an ABI 3100 automated sequencer (Applied Biosystems, Foster City, CA), and determined the genetic identity of the species based on alignment of the nucleotide sequences of the *cox*1 gene [19].

## Results

**Fig. 1:**
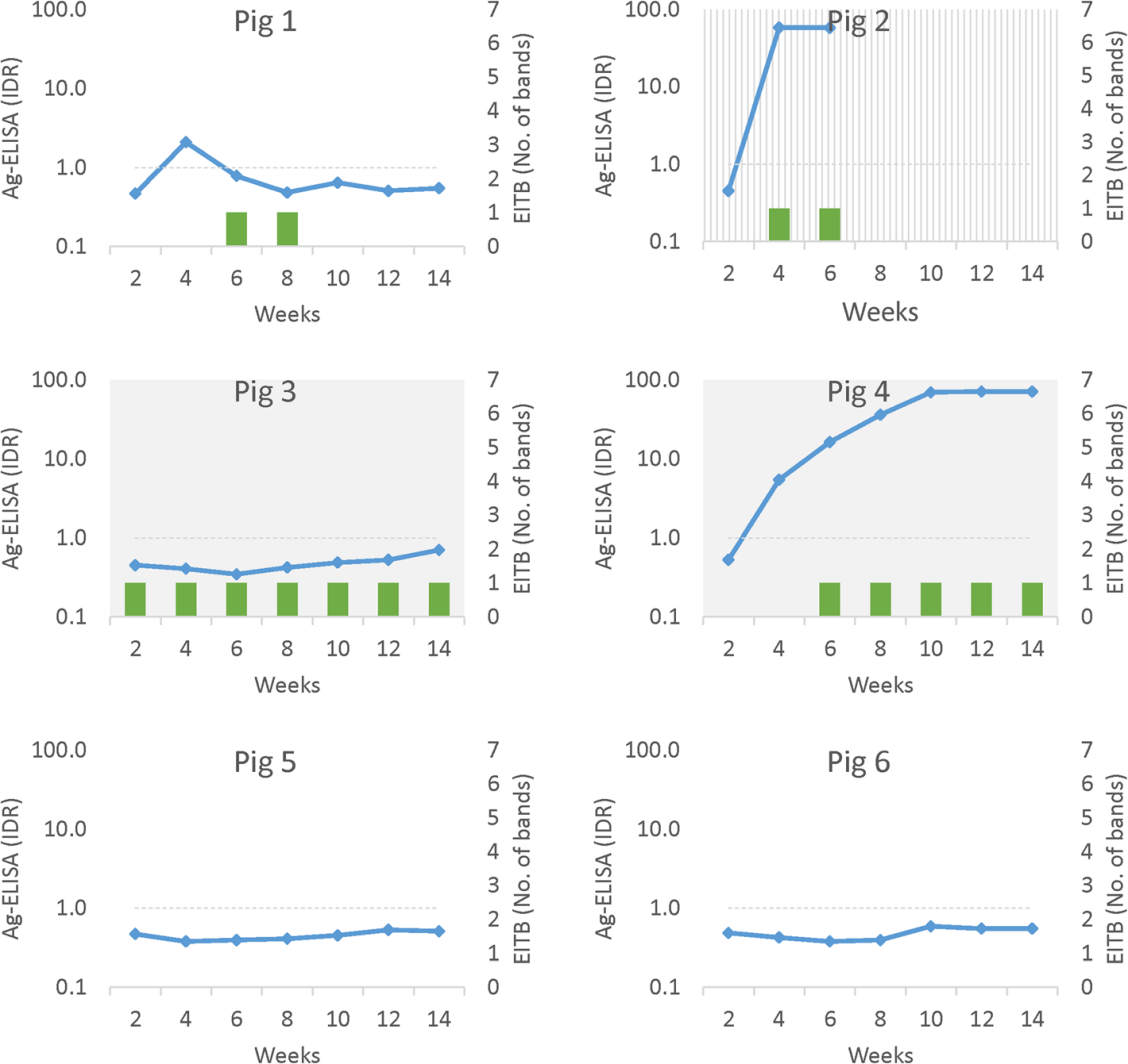
Serology and necropsy outcomes for pigs exposed to *Taenia hydatigena via* oral challenge with proglottids

**Fig. 2:**
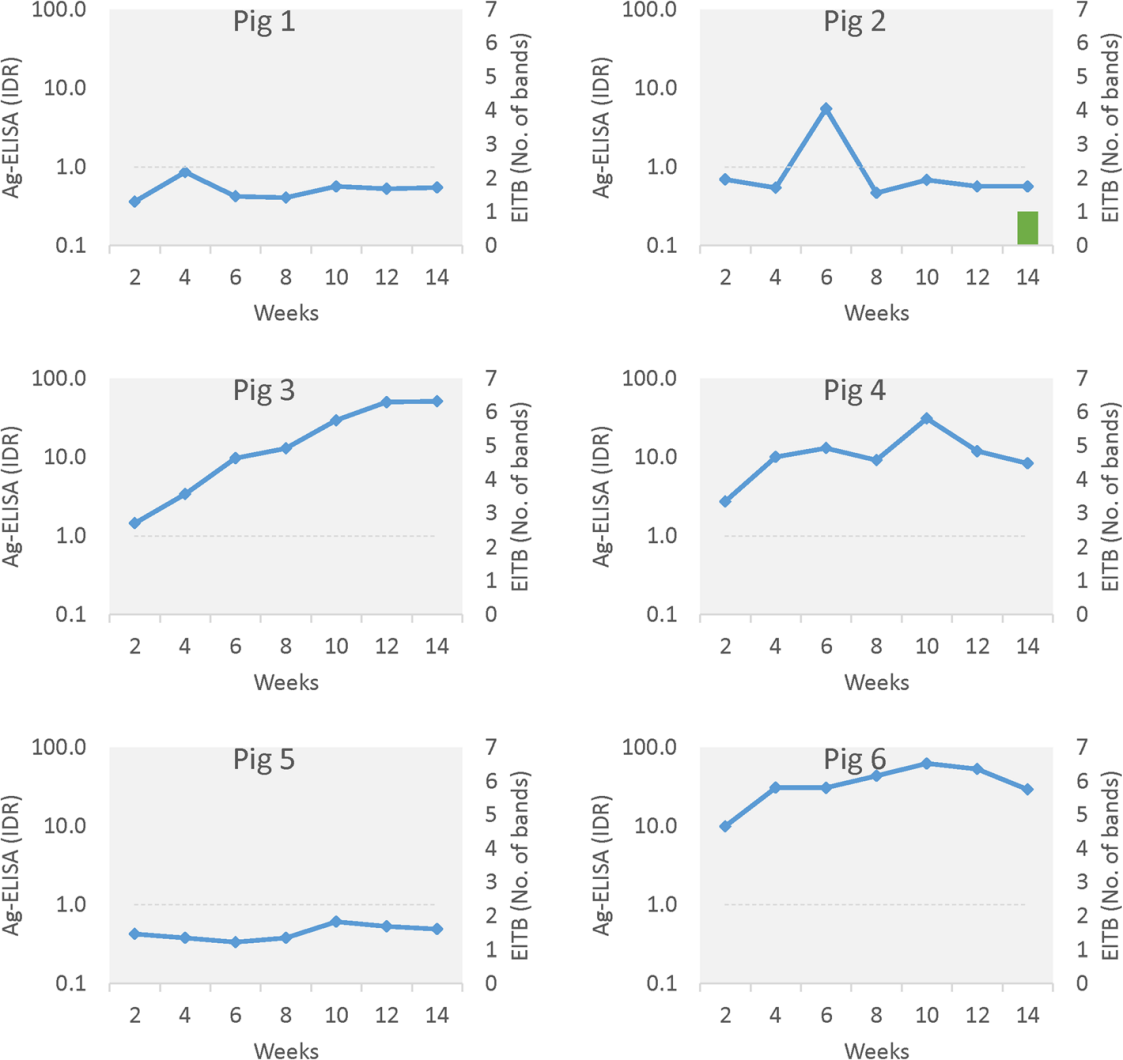
Serology and necropsy outcomes for pigs exposed to *Echinococcus granulosus via* oral challenge with proglottids

Oral challenge resulted in metacestode infections in both groups; 2 out of 6 pigs in the *T. hydatigena* group and 6 out of 6 pigs in the *E. granulosus* group had visible metacestode infection at time of necropsy, all confirmed by molecular diagnosis to be the corresponding species. Circulating Ag was present in some, but not all, pigs in both groups; three pigs with verified infection (1 in *T. hydatigena* group and 2 in *E. granulosus* group) were negative on the Ag test. The presence of circulating Ag or anti-glycoprotein Abs over time for all pigs is shown in Figs. 1 and 2 (corresponding data are available in Additional file 1: Tables S1 and S2).

In the *T. hydatigena* group, four of the six pigs became seropositive on the LLGP EITB test, and in all instances, the seropositive reaction was only against the GP50 band. GP50 seropositivity first appeared between 2–6 weeks after exposure and was persistent at all subsequent testing points for two pigs (Pigs 3 and 4), both of which were infected with *T. hydatigena* cysts upon necropsy. Pig 1 had a transient seropositive reaction against GP50 which followed a transient peak of circulating Ag; no cysts were found in this pig on necropsy. Pig 2 rapidly showed both circulating Ag and reactivity against GP50, but it died 6 weeks after exposure so serological trends could not be fully evaluated; no cysts were found on necropsy despite a high level of circulating Ag. The final two pigs (Pigs 5 and 6) remained seronegative for Ag and Ab and no cysts were detected in either upon necropsy.

In the *E. granulosus* group all six pigs developed viable cyst infections detected at necropsy. One pig (Pig 2) developed a seropositive reaction against GP50 but only in a single serum sample. This seropositive reaction developed approximately six weeks after a transient peak in circulating Ag. In the other 5 pigs there was no Ab response on LLGP EITB at any testing point. Four out of the six *E. granulosus*-infected pigs had circulating Ag present; three (pigs 3, 4 and 6) showed persistently high or increasing Ag levels, one (pig 2) had transient circulating Ag, and two (pigs 1 and 5) did not develop circulating Ag.

## Discussion

**Fig. 3:**
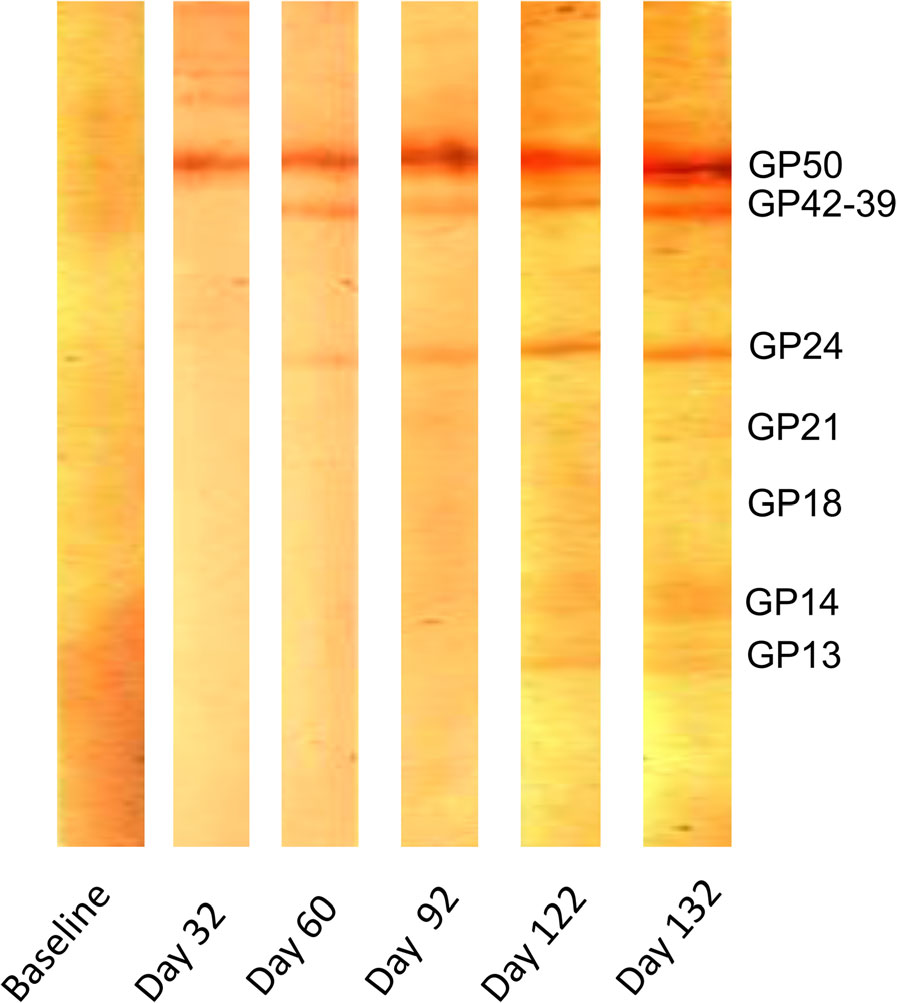
Serial EITB LLGP test strips showing progressive development of antibodies against the glycoprotein antigens (bands) present (Garcia et al., unpublished data)

The results of this study confirm that exposure of pigs to *T. hydatigena* can result in cross-reaction against the GP50 diagnostic band of the LLGP EITB. Reaction against GP50 was seen in four out of six pigs challenged with *T. hydatigena* with Abs appearing as soon as two weeks post-infection. A few different Ab patterns were observed, including transient Ab associated with apparently cleared infection, persistent Ab in established infection, and a lack of Ab in both infected and apparently healthy animals. Although an Ab response against GP50 was also observed in the group of pigs challenged with *E. granulosus*, the evidence for cross-reaction is not conclusive, as the positive result was seen in only one out of 42 samples from the six infected pigs in this group. The results of the LLGP EITB for both *T. hydatigena* and *E. granulosus*-exposed pigs clearly contrast with those that emerge after oral challenge of pigs with *T. solium* [10]. While Abs against GP50 emerge shortly after challenge with *T. solium*, a steady progression of circulating Abs against the other diagnostic bands follows over ensuing weeks, with reactions of up to seven bands occurring in established cyst infection (Fig. 3).

The LLGP EITB has been considered the gold standard for pig population studies of *T. solium* for decades given its high reported sensitivity and specificity [10]. However, the observed cross-reaction at GP50 suggests that LLGP EITB results should be interpreted with caution in pigs, particularly in free-roaming animals that are exposed through their foraging to a broad range of parasites. However, we strongly feel that the LLGP EITB still provides useful information regarding the distribution and transmission of *T. solium*, and that use of the test for this purpose should not be discarded. Of the seven glycoprotein antigens that were included in the assay, only GP50 has been shown to cross-react with other organisms. Excluding GP50 from the assay will address the known cross-reactivity and improve the specificity of the assay, which can then be interpreted as positive if there is reactivity against any of the other six diagnostics bands. This approach will lower the sensitivity of the assay since GP50 is typically the first band to appear upon exposure to *T. solium*, therefore recent exposures may be missed. However, we view this as an acceptable trade-off given the otherwise exquisite sensitivity of the EITB LLGP and the need to maximize specificity for the intended use. Similar experiments involving oral challenge with other parasites should be conducted to support this approach. It can be noted, however, that a recent study found no cross-reaction on any band after oral challenge of pigs with *T. saginata* eggs [12].

The LLGP EITB has been criticized based on the observation that the positive predictive value of seropositivity is around 50% in necropsies of naturally-infected pigs [11]. Some of this can be attributed to the cross-reaction at GP50. However, it is important to stress that this criticism is based on an incorrect interpretation of what the assay delivers. The LLGP EITB is an Ab detection test, and as such it is expected to be positive in pigs that are exposed to *T. solium* eggs but do not develop established infection, as well as in pigs in which infection occurs but is cleared by the host immune response. These Abs continue to circulate after the infection has been cleared and thereby contribute to the host’s acquired immunity. The results of the LLGP EITB therefore, should not be expected to accurately distinguish between active or cleared infection on an individual pig level. The results of the LLGP EITB can be correctly interpreted as a measure of the frequency of exposure to *T. solium* oncospheres in a population of pigs. Interpreted in this way, the LLGP EITB provides information that can be used to monitor the transmission of *T. solium* in a population of pigs.

The World Health Organization recently published results of a stakeholder meeting in which the optimal and minimal characteristics for tests of *T. solium* infection in human and pig hosts were proposed [20]. Per the published Target Product Profiles (TPPs), the primary intended use of a test for porcine cysticercosis would be to monitor *T. solium* control interventions. It is our view that that LLGP EITB can provide information for this purpose. However, we readily agree that the LLGP EITB has deficiencies that keep it from being an ideal test for this purpose. As previously discussed, it falls short of the TPP requirement to test positive only in the presence of viable cysts and to revert to negative within ten weeks after the cyst infection is cleared. The LLGP EITB has other deficiencies as well, such as the limited availability of the test due to the requirement for native cysts and expensive equipment for preparation of the reagents, as well as a qualitative format that results in variability in interpretation. The availability of synthetic and recombinant forms of all of the antigens used on the strip may solve the former issue [21–23], although an alternate platform such as multiplexing would be required to produce a quantitative result.

Until an alternative test that fulfills all of performance characteristics detailed in the published TPP’s is available, we will continue to use the EITB LLGP to monitor progress of control interventions. We will, however, exclude GP50 and instead consider reactivity against any of the other six glycoprotein bands as a positive result. Currently available Ag-detection assays cross-react broadly at the genus level and therefore do not have the specificity required to monitor transmission [16, 24]. Although detailed necropsy has been suggested as an alternative approach, this is highly resource-intensive and impractical in most settings [11, 25]. The sensitivity of necropsy is also unknown, and it is likely that some pigs with just one or a few cysts may be miscategorized as uninfected. Given that many naturally infected pigs have very few cysts in the entire carcass [6, 25–27], necropsy can be expected to routinely underestimate transmission on the population level. Tongue examination for cysts is similarly afflicted with suboptimal sensitivity for the purpose of monitoring transmission, although it can be employed to identify focal points of transmission [5, 26].

An additional finding of this study was confirmation that the B158/B60 monoclonal antibody ELISA cross-reacts with *E. granulosus* in pigs. Four of six pigs challenged with *E. granulosus* proglottids demonstrated transient or persistent positive results indicating the presence of circulating Ags. This finding could have potential implications for use of the assay in humans, as there is no reason to suspect that B158/B60 would not also result in cross-reactions in humans. Previously, the specificity of Ag-detection tests in humans has not been considered to be a particularly relevant given the lack of other important metacestode infections in humans. Since Ag-detection using B158/B60 is routinely used to support diagnosis of NCC and to monitor the progress of the infection after treatment, the potential for cross-reactions of this assay in humans should be evaluated.

This study also has limitations which should be considered in the interpretation of the results. We only evaluated *T. hydatigena* and *E. granulosus* in our experiments, so we cannot rule out the possibility of cross-reactions on the LLGP EITB for other parasites, at the GP50 or other diagnostic bands. Similarly, we cannot rule out the possibility of reactions to other bands developing in later stages of infection with *T. hydatigena* and *E. granulosus*, given that we monitored serology only until 14 weeks post-exposure before conducting necropsy. It is also possible that the circulating Ab and Ag we observed are limited to the early period of infection and could wane in later stages of infection.

## Conclusions

Serological assays that measure the presence of Ab or Ag in pig populations are important tools that allow efficient and timely monitoring of the transmission of *T. solium* cysticercosis, particularly in the context of ongoing control interventions. However, all available serological assays have limitations that fall short of the ideal characteristics identified by the WHO and other stakeholders [20]. The study presented here confirms that *T. hydatigena* cross-reacts at the GP50 diagnostic band of the EITB LLGP assay in pigs, and that the results of this assay in pigs should be interpreted with caution. Until an alternative method that fulfills all of the performance criteria in the TPP becomes available, it is our view that the EITB LLGP can still be used to monitor transmission in populations of pigs by excluding GP50, and instead relying on the presence/absence of the other six diagnostic glycoprotein bands.

## Additional file

Additional file 1: **Table S1.** Serology and necropsy outcomes for pigs experimentally infected with *Taenia hydatigena*. **Table S2.** Serology and necropsy outcomes for pigs experimentally infected with *Echinococcus granulosus*. (PDF 302 kb)

## References

[1] Ndimubanzi PC, Carabin H, Budke CM, Nguyen H, Qian Y-J, Rainwater E (2010). A systematic review of the frequency of neurocyticercosis with a focus on people with epilepsy. PLoS Negl Trop Dis..

[2] Accelerating work to overcome the global impact of neglected tropical diseases: A roadmap for implementation. http://www.who.int/neglected_diseases/NTD_RoadMap_2012_Fullversion.pdf. Accessed 10 Dec 2018.

[3] Gonzalez AE, Gilman RH, Garcia HH, McDonald J, Kacena K, Tsang V (1994). Use of sentinel pigs to monitor envionmental *Taenia solium* contamination. The Cysticercosis Working Group in Peru (CWG). Am J Trop Med Hyg..

[4] Garcia HH, Gilman RH, Gonzalez AE, Verastegui M, Rodriguez S, Gavidia C (2003). Hyperendemic human and porcine *Taenia solium* infection in Perú. Am J Trop Med Hyg..

[5] O’Neal SE, Moyano LM, Ayvar V, Rodriguez S, Gavidia C, Wilkins PP (2014). Ring-screening to control endemic transmission of *Taenia solium*. PLoS Negl Trop Dis..

[6] Gavidia CM, Verastegui MR, Garcia HH, Lopez-Urbina T, Tsang VCW, Pan W (2013). Relationship between serum antibodies and *Taenia solium* larvae burden in pigs raised in field conditions. PLoS Negl Trop Dis..

[7] Mohan VR, Tharmalingam J, Muliyil J, Oommen A, Dorny P, Vercruysse J (2013). Prevalence of porcine cysticercosis in Vellore, South India. Trans R Soc Trop Med Hyg..

[8] Sikasunge CS, Phiri IK, Phiri AM, Siziya S, Dorny P, Willingham AL (2008). Prevalence of *Taenia solium* porcine cysticercosis in the eastern, southern and western provinces of Zambia. Vet J..

[9] Tsang VCW, Brand JA, Boyer AE (1989). An enzyme-linked immunoelectrotransfer blot assay and glycoprotein antigens for diagnosing human cysticercosis (*Taenia solium*). J Infect Dis..

[10] Tsang VCW, Pilcher JA, Zhou W, Boyer AE, Kamango-Sollo EIO, Rhoads ML (1991). Efficacy of the immunoblot assay for cysticercosis in pigs and modulated expression of distinct IgM/ IgG activities to *Taenia solium* antigens in experimental infections. Vet Immunol Immunopathol..

[11] Lightowlers MW, Garcia HH, Gauci CG, Donadeu M, Abela-Ridder B. Monitoring the outcomes of interventions against *Taenia solium*: options and suggestions. Parasite Immunol. 2016;38:158–69. 10.1111/pim.12291 PMC4819694 26538513

[12] Dorny P, Dermauw V, Van Hul A, Trevisan C, Gabriël S (2017). Serological diagnosis of *Taenia solium* in pigs: no measurable circulating antigens and antibody response following exposure to *Taenia saginata* oncospheres. Vet Parasitol..

[13] Garcia HH, Gonzalez AE, Tsang VCW, O’Neal SE, Llanos-Zavalaga F, Gonzalvez G (2016). Elimination of *Taenia solium* transmission in northern Peru. N Engl J Med..

[14] Muro C, Gomez-Puerta LA, Flecker RH, Gamboa R, Barreto PV, Dorny P (2017). Porcine cysticercosis: possible cross-reactivity of *Taenia hydatigena* to GP50 antigen in the enzyme-linked immunoelectrotransfer blot assay. Am J Trop Med Hyg..

[15] Gemmell MA (1973). Surveillance of *Echinococcus granulosus* in dogs with arecoline hydrobromide. Bull World Health Organ..

[16] Brandt JRA, Falla N, Eulemans F, Brijs L (1992). Antibody-based ELISA for the detection of excretory-secretory antigens in *Taenia saginata* cysticercosis. Int J Parasitol..

[17] Liu G-H, Lin R-Q, Li M-W, Liu W, Liu Y, Yuan Z-G (2011). The complete mitochondrial genomes of three cestode species of *Taenia* infecting animals and humans. Mol Biol Rep..

[18] Bowles J, McManus DP (1994). Genetic characterization of the Asian *Taenia*, a newly described taeniid cestode of humans. Am J Trop Med Hyg..

[19] Zhang G, Chen J, Yang Y, Liu N, Jiang W, Gu S (2014). Utility of DNA barcoding in distinguishing species of the family Taeniidae. J Parasitol..

[20] Donadeu M, Fahrion AS, Olliaro PL, Abela-Ridder B (2017). Target product profiles for the diagnosis of *Taenia solium* taeniasis, neurocysticercosis and porcine cysticercosis. PLoS Negl Trop Dis..

[21] Hancock K (2004). Characterization and cloning of GP50, a *Taenia solium* antigen diagnostic for cysticercosis. Mol Biochem Parasitol..

[22] Hancock K, Khan A, Williams FB, Yushak ML, Pattabhi S, Noh J (2003). Characterization of the 8-kilodalton antigens of *Taenia solium* metacestodes and evaluation of their use in an enzyme-linked immunosorbent assay for serodiagnosis. J Clin Microbiol..

[23] Hancock K, Pattabhi S, Whitfield FW, Yushak ML, Lane WS, Garcia HH (2006). Characterization and cloning of T24, a *Taenia solium* antigen diagnostic for cysticercosis. Mol Biochem Parasitol..

[24] Harrison LJS, Joshua GWP, Wright SH, Parkhouse RME (1989). Specific detection of circulating surface/secreted glycoproteins of viable cysticerci in *Taenia saginata* cysticercosis. Parasite Immunol..

[25] Lightowlers MW, Assana E, Jayashi CM, Gauci CG, Donadeu M (2015). Sensitivity of partial carcass dissection for assessment of porcine cysticercosis at necropsy. Int J Parasitol..

[26] Flecker RH, Pray IW, Santivaňez SJ, Ayvar V, Gamboa R, Muro C (2017). Assessing ultrasonography as a diagnostic tool for porcine cysticercosis. PLoS Negl Trop Dis..

[27] Sciutto E, Martínez JJ, Villalobos NM, Hernández M, José MV, Beltrán C (1998). Limitations of current diagnostic procedures for the diagnosis of *Taenia solium* cysticercosis in rural pigs. Vet Parasitol..

