# Supplementary material for: Evaluation of cross-reactivity to Taenia hydatigena and Echinococcus granulosus in the enzyme-linked immunoelectrotransfer blot assay for the diagnosis of porcine cysticercosis
Source: Parasit Vectors. 2019 Jan 24;12:57. doi: 10.1186/s13071-018-3279-5 (PMC6346519; doi:10.1186/s13071-018-3279-5)
Supplement: Supplementary file 1 — Table S1. Serology and necropsy outcomes for pigs experimentally infected with Taenia hydatigena. Table S2. Serology and necropsy outcomes for pigs experimentally infected with Echinococcus granulosus. (PDF 302 kb) [file 13071_2018_3279_MOESM1_ESM.pdf]

Supplemental Table 1. Serology and necropsy outcomes for pigs experimentally infected with *Taenia hydatigena*.

**Pig Id.**	**Outcome**	**Time post-infection (weeks)**						
		2	4	6	8	10	12	14
**1**	LLGP EITB	0	0	**1**	**1**	0	0	0
	Ag-ELISA	0.5	**2.1**	0.8	0.5	0.6	05	0.5
	Necropsy	-	-	-	-	-	-	0
**2**	LLGP EITB	0	**1**	**1**	-	-	-	-
	Ag-ELISA	0.5	**58.2**	**58.0**	-	-	-	-
	Necropsy	-	-	0	-	-	-	-
**3**	LLGP EITB	**1**	**1**	**1**	**1**	**1**	**1**	**1**
	Ag-ELISA	0.5	0.4	0.3	0.4	0.5	0.5	0.7
	Necropsy	-	-	-	-	-	-	11
**4**	LLGP EITB	0	0	**1**	**1**	**1**	**1**	**1**
	Ag-ELISA	0.5	**5.4**	**16.3**	**36.2**	**69.8**	**71.4**	**71.1**
	Necropsy	-	-	-	-	-	-	4
**5**	LLGP EITB	0	0	0	0	0	0	0
	Ag-ELISA	0.5	0.4	0.4	0.4	0.5	0.5	0.5
	Necropsy	-	-	-	-	-	-	0
**6**	LLGP EITB	0	0	0	0	0	0	0
	Ag-ELISA	0.5	0.4	0.4	0.4	0.6	0.5	0.5
	Necropsy	-	-	-	-	-	-	0

EITB: Number of reactive bands on enzyme-linked immunoelectrotranfer blot. All positive EITB LLGP results were reactive only to the GP50 band only. **Ag-ELISA:** Optical density ratio on enzyme-linked immunosorbent assay. Values ≥1 are considered positive. **Necropsy**: Number of parasite cysts found during fine dissection of animal carcass.

Supplemental Table 2. Serology and necropsy outcomes for pigs experimentally infected with *Echinococcus granulosus*.

**Pig Id.**	**Outcome**	**Time post-infection (weeks)**						
		2	4	6	8	10	12	14
**1**	LLGP EITB	0	0	0	0	0	0	0
	Ag-ELISA	0.4	0.9	0.4	0.4	0.6	0.5	0.5
	Necropsy	-	-	-	-	-	-	3
**2**	LLGP EITB	0	0	0	0	0	0	**1**
	Ag-ELISA	0.7	0.5	5.5	0.5	0.7	0.6	0.6
	Necropsy	-	-	-	-	-	-	2
**3**	LLGP EITB	0	0	0	0	0	0	0
	Ag-ELISA	**1.4**	**3.4**	**9.7**	**12.9**	**29.5**	**50.2**	**51.4**
	Necropsy	-	-	-	-	-	-	1
**4**	LLGP EITB	0	0	0	0	0	0	0
	Ag-ELISA	**2.7**	**10.1**	**13.0**	**9.2**	**31.1**	**11.9**	**8.3**
	Necropsy	-	-	-	-	-	-	1
**5**	LLGP EITB	0	0	0	0	0	0	0
	Ag-ELISA	0.4	0.4	0.3	0.4	0.6	0.5	0.5
	Necropsy	-	-	-	-	-	-	2
**6**	LLGP EITB	0	0	0	0	0	0	0
	Ag-ELISA	**9.9**	**30.8**	**30.7**	**43.5**	**62.5**	**53.3**	**29.3**
	Necropsy	-	-	-	-	-	-	1

EITB: Number of reactive bands on enzyme-linked immunoelectrotranfer blot. All positive EITB LLGP results were reactive only to the GP50 band only. **Ag-ELISA:** Optical density ratio on enzyme-linked immunosorbent assay. Values ≥1 are considered positive. **Necropsy**: Number of parasite cysts found during fine dissection of animal carcass.
